# Effect of Different Substrates on Soil Microbial Community Structure and the Mechanisms of Reductive Soil Disinfestation

**DOI:** 10.3389/fmicb.2019.02851

**Published:** 2019-12-11

**Authors:** Xingyan Tan, Hongkai Liao, Liangzuo Shu, Huaiying Yao

**Affiliations:** ^1^Ningbo Key Lab of Urban Environment Process and Pollution Control, Ningbo Urban Environment Observation and Research Station, Institute of Urban Environment, Chinese Academy of Sciences, Ningbo, China; ^2^School of Life Sciences, Huaibei Normal University, Key Laboratory of Plant Resources and Biology of Anhui Province, Huaibei, China; ^3^Key Lab of Urban Environment and Health, Institute of Urban Environment, Chinese Academy of Sciences, Xiamen, China; ^4^Research Center for Environmental Ecology and Engineering, Wuhan Institute of Technology, Wuhan, China

**Keywords:** reductive soil disinfestation, tomato, organic matter, C/N ratio, soil microbial community

## Abstract

Reductive soil disinfestation (RSD) has recently attracted much attention owing to its effectiveness for controlling pathogens. In this study, we aimed to evaluate the effects of different C/N substrates on RSD and to explore the changes in microbial community structure during RSD treatment. The experimental set up included 10 groups, as follows: CK, without substrates; RSD treatments with alfalfa (*Medicago sativa L.*)[AL], maize (*Zea mays Linn. Sp.*) straw [MS], and rice (*Oryza sativa* L.) straw [RS], with three levels of addition (0.5% [L], 2% [M], and 5% [H]), yielding ALL, ALM, ALH, MSL, MSM, MSH, RSL, RSM, and RSH groups. Compared with CK, RSD treatments significantly increased the content of ${\text{NH}}_{4}^{+}$-N, and effectively eliminated the accumulated ${\text{NO}}_{3}^{-}$-N in the soil. The relative abundances of organic acid producers, including *Clostridium*, *Coprococcus*, and *Oxobacter*, in all RSD groups were significantly higher than those in the CK by day 21. Moreover, on day 21, *Aspergillus* and *Fusarium* in all RSD groups were significantly lower than those in the CK. In summary, RSD treatments clearly increased the relative abundances of organic acid generators and effectively inhibited pathogens; however, when the C/N was too low and the amount of addition too high, ammonia poisoning and rapid growth of some microorganisms (e.g., *Pseudallescheria* and *Arthrographis*) may occur.

## Introduction

Tomatoes are a global economic crop and China is the leading producer of tomatoes worldwide. However, as the cultivation period increases, accumulation of soil-borne plant pathogens in the soil also increases (Fu et al., 2017). These pathogens can survive in the soil for long periods, thereby impairing plant growth. Traditional chemical fungicides have been phased out because of increasing concerns regarding sustainable development of agriculture and human health (Butler et al., 2012; Huang et al., 2016). Therefore, non-chemical soil sterilization methods, including reductive soil disinfestation (RSD) have been rapidly developed.

Reductive soil disinfestation refers to the addition of easily decomposable organic matter to the soil, saturation of the soil with water, and then covering of the soil with a plastic film and incubation at a high temperature for 3–4 weeks; this kills pathogens by creating anaerobic reducing conditions (Momma et al., 2013; Butler et al., 2014). This method was developed in the Netherlands in the year 2000 and has now been applied to many crop production systems (Blok et al., 2000), including tomatoes (Di Gioia et al., 2017; Guo et al., 2018), potatoes (Messiha et al., 2007), strawberries (Shennan et al., 2014), and watermelons (Liu et al., 2018), which are prone to the problems associated with continuous cropping. RSD has significant effects on inhibition of soil-borne diseases. For example, in an anaerobic environment, some aerobic pathogens will not survive (Serrano-Pérez et al., 2017). Additionally, the organic acids produced during the decomposition of organic matter can effectively kill some soil-borne plant pathogens, including *Fusarium oxysporum* (Butler et al., 2012; Huang et al., 2014; Meng et al., 2017). Significant changes in the microbial community structure have also been noted after RSD treatment (Huang et al., 2016; Guo et al., 2018; Mazzola et al., 2018), and rapid growth of anaerobic bacteria can lead to decreases in aerobic pathogens.

The choice of organic matter is important for disease control under anaerobic conditions. RSD uses easily decomposable organic matter, such as plant residues, diluted ethanol, molasses, or manure (Di Gioia et al., 2017; Zhao et al., 2018). The application of different organic materials in RSD treatment may result in different effects. Wen et al. (2015) found that application of maize straw in RSD treatment can effectively inhibit *Artemisia selengensis* root rot pathogens and the inhibition efficiency can reach 90% when the application rate is 2%. The combined application of molasses and composted poultry litter has been shown to have strong effects on inhibiting fungi and nematodes (Rosskopf et al., 2014). Additionally, the separate use of molasses can also suppress *F. oxysporum* (Butler et al., 2012). Wheat bran used as the carbon source in RSD can control *Fusarium* wilt by reducing the viability of *F. oxysporum* f. sp. *lycopersici* chlamydospores (Momma et al., 2006; Mowlick et al., 2012). Treatment with crop straw has also been explored in China (Gadde et al., 2009; Li et al., 2018).

Examining the effects of substrate input amount on pathogens may facilitate optimization of the application rate of organic matter (Wen et al., 2015). Although previous studies have shown that greater input of organic material increases pathogen inhibition (Blok et al., 2000), the amount of organic material added needs to be controlled based on the growth of crops in the soil. Additionally, the amount of soil-borne pathogens can be significantly reduced, and the community structure of microorganisms can be clearly changed, by using RSD to treat continuous cropping soil. However, few studies have evaluated the specific trends in microbial community structure during soil treatment. Therefore, in this study, we aimed to investigate the effects of different C/N substrates on RSD and explore specific changes in the community structure of bacteria and fungi by RSD during soil treatment and after tomato planting.

## Materials and Methods

### Soil Sampling and Experimental Design

The soils used in this test were from a greenhouse located in Changzhou, Jiangsu Province (31°55′, 119°51′), East China. The soils were rotated for tomato cultivation for the past 4 years, and soil samples were collected after the tomatoes were harvested. Physical and chemical properties of soils were as follows: pH 6.60; total N 1.47 g kg^–1^; total P 0.66 g kg^–1^; total K 9.06 g kg^–1^; ${\text{NH}}_{4}^{+}$-N 25.47 mg kg^–1^; ${\text{NO}}_{3}^{-}$-N 473.41 mg kg^–1^; and organic matter 21.94 g kg^–1^.

Pots (15 cm × 15 cm) were filled with 2 kg soil, and treatments were set up as follows: (1) CK, without substrate; (2) ALL, RSD with 0.5% alfalfa [AL] (*Medicago sativa L.*) (substrate/soil ratio [w/w], the same below); (3) ALM, RSD with 2% AL; (4) ALH, RSD with 5% AL; (5) MSL, RSD with 0.5%maize straw [MS] (*Zea mays Linn. Sp.*); (6) MSM, RSD with 2% MS; (7) MSH, RSD with 5%MS; (8) RSL, RSD with 0.5% rice straw [RS] (*Oryza sativa* L.); (9) RSM, RSD with 2% RS; (10) RSH, RSD with 5% RS. The C/N ratios of the three substrates used in the test are shown in Table 1. Each treatment included three replicates. All treatments were cultured at 35°C for 21 days under flooding and covered with transparent plastic film (Thickness = 0.12 mm). Sampling was performed on days 7, 14, and 21. After 21 days, the plastic films were removed, and the soils in all treatments were drained for 1 week and dried. Next, 21-day-old tomato seedlings, each with the same growth, were selected and transplanted into the pots. The tomato plants were incubated in a walk-in incubator, with average day and night temperatures of 30 and 20°C, respectively, during the tomato growth period. Moreover, the soils were equally amended with urea (100 mg N kg^–1^) and KH_2_PO_4_ (100 mg K kg^–1^) for all treatments. The tomato plants were harvested after 60 days, and rhizosphere soil samples were collected after planting.

**TABLE 1:** Nutrient contents of maize, rice straw, and alfalfa.

	**Total N (g kg^–1^)**	**Total C (g kg^–1^)**	**C:N ratio**
Alfalfa (AL)	31.51	455.80	14.47
Maize straw (MS)	13.48	456.33	33.80
Rice straw (RS)	5.87	404.78	68.61

### Soil Characteristics and Plant Nutrient Analysis

Soil pH was measured in a 1:2.5 (v/v) soil/water ratio using an Accumet XL600 pH meter (Fisher Scientific, Inc., United States). Soil dissolved organic carbon (DOC) was extracted with 0.5M K_2_SO_4_ at a soil/solution ratio of 1:5 and detected using a TOC-VCPH/CPN analyzer (Shimadzu Inc., Japan). Soil ${\text{NO}}_{3}^{-}$-N and ${\text{NH}}_{4}^{+}$-N was extracted with 1M KCl at a soil/solution ratio of 1:10 on a shaker for 60 min at 250 rpm at 25°C. The extract was filtered, and the concentrations of ${\text{NO}}_{3}^{-}$-N and ${\text{NH}}_{4}^{+}$-N were determined with a continuous-flow analyzer (SAN + +; Skalar, Breda, Holland).

### DNA Extraction and Real-Time Quantitative Polymerase Chain Reaction (qPCR)

A FastDNA Spin Kit for Soil (MP Biomedicals, Cleveland, OH, United States) was used for extraction of DNA from 0.5 g soil samples according to the manufacturer’s recommendations. Next, the quality and concentration of DNA were detected using a NanoDrop 2000 spectrophotometer (Thermo, Waltham, MA, United States). The DNA samples were stored in a −20°C refrigerator for subsequent analysis.

qPCR amplification was performed on a Light-Cycler Roche 480 instrument (Roche Molecular Systems, Switzerland). The reaction system had a total volume of 20 μL, including 10 μL Go Taq qPCR master mix (Promega, Madison, WI, United States), 0.4 μL forward and reverse primers (10 μM), and 1 μL template DNA. The abundances of bacteria and fungi were determined using the 16S rRNA geneV4–V5 and ITS1 region respectively, and using the primer pair 515F (GTGCCAGCMGCCGCGG)/907R (CCGTCAATTCMTTTRAGTTT) and ITS1F (CTTGGTCATT TAGAGGAAGTAA)/ITS2R (GCTGCGTTCTTCATCGATGC), respectively. The conditions for bacterial 16S rRNA gene qPCR amplification were based on the protocol described by Long et al. (2018). The thermal cycling conditions for the fungal internal transcribed spacer (ITS) gene were as follows: an initial denaturation step at 95°C for 5 min; followed by 45 cycles of 95°C for 15 s, 55°C for 40 s, 72°C for 45 s, and 84°C for 15 s (fluorescence intensity detection); a final extension at 72°C for 60 s; and finally, melting curve analysis. All standard curves were prepared by a 10-fold gradient dilution method for environmental sample plasmid DNA, and the R^2^ values of the standard curves of bacteria and fungi were greater than 0.9992 and 0.9965, respectively. The amplification efficiencies of qPCR for bacteria and fungi were greater than 95% and 90%, respectively.

### Illumina HiSeq2500 Sequencing and Data Processing

In total, 120 DNA samples (10 treatments × three replicates × four time points) were selected for HiSeq. DNA samples were subjected to sequencing at Biotechnologies, Inc. (Beijing, China) on an Illumina HiSeq instrument (United States). The bacterial 16S rRNA gene V4–V5 region was amplified using 515F and the individual barcode reverse primer 907R (Xu et al., 2012; Long et al., 2016), and ITS1F and the individual barcode reverse primer ITS2R (Huang et al., 2016) were used to amplify the fungal ITS1 region. The results of high-throughput sequencing have been uploaded to NCBI (SRA accession: PRJNA523309).

The default settings of the Quantitative Insights into Microbial Ecology (QIIME 1.9.1) platform were used for analysis of bacterial 16S rRNA genes and fungal ITS genes (Caporaso et al., 2010). Only the targeting sequence was retained in subsequent analyses. Operational taxonomic units (OTUs) were identified with a cutoff of 97% similarity using RDP classifier (Claesson et al., 2009). The annotation databases for 16S rRNA gene sequencing data and fungal ITS region sequencing data are the Greengenes database (13.8) and the UNITE database (12.11), respectively. For bacteria, the sampling depth for rarefaction curve analysis was optimized to 21590 sequences for soil DNA samples during RSD treatments and 26911 sequences for soil DNA samples after tomato planting. For fungi, the sampling depth for rarefaction curve analysis was optimized to 15118 sequences for soil DNA samples during RSD treatments and 3402 sequences for soil DNA samples after tomato planting.

### Community Data and Statistical Analysis

The rarefied OTU table for each treatment was used to calculate the microbial community alpha-diversity. Principal coordinate analysis (PCoA) was performed based on both weighted_unifrac and Bray_Curtis distance matrices in bacteria and fungi, respectively, to visualize the pairwise community dissimilarities between samples. Duncan tests (*P* < 0.05) in IBM SPSS 20.0 (SPSS, Inc., Chicago, IL, United States)were used to test significant differences among treatments by one-way analysis of variance, and the significant differences in soil microbial community structures have been analyzed using PERMANOVA.

## Results

### Changes in Soil Properties During RSD Treatment

**FIGURE 1:**
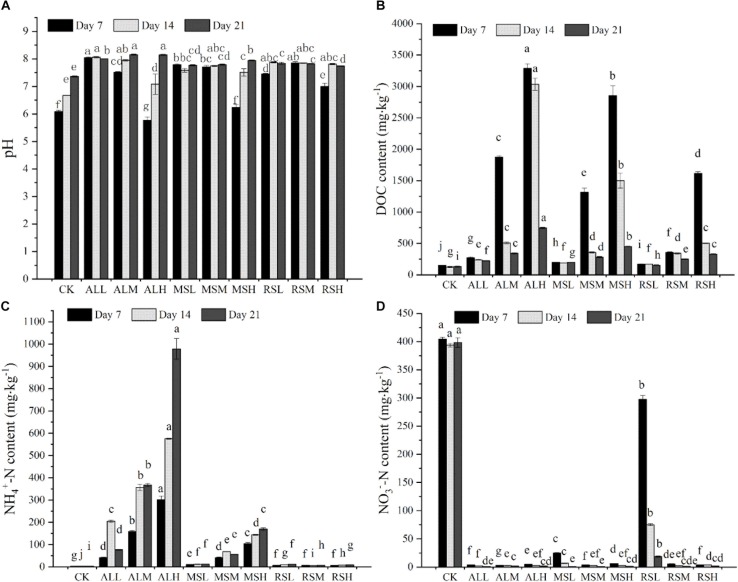
The soil pH **(A)**, DOC content **(B)**, ammonia nitrogen content **(C)**, and nitrate nitrogen content **(D)** during RSD. Bars with different letters represent significant differences among the 10 treatments at the same time, as determined using Duncan’s tests (*P* < 0.05). Error bars indicate standard errors.

Compared with the CK group, on days 14 and 21, the pH of all RSD treatments was clearly increased. At the end of incubation, the pH values were highest in the ALH and ALM groups (Figure 1A). Input of organic matter increased the content of soluble DOC in the soil. For the same substrate treatment, the content of DOC increased as the amount added increased. Moreover, over time, the DOC content in each group gradually decreased (Figure 1B). On days 7, 14, and 21, sampling results indicated that the ${\text{NH}}_{4}^{+}$-N content in all RSD treatments was higher than that in the CK group. Following treatment with MS and AL, the ${\text{NH}}_{4}^{+}$-N content increased as the amount of substrate added increased over time. At the end of the RSD, the ${\text{NH}}_{4}^{+}$-N content in the ALH group reached up to 978.8 mg kg^–1^ (Figure 1C). However, throughout the entire soil treatment period, the ${\text{NH}}_{4}^{+}$-N content in the rice straw treatment group was maintained at a lower concentration at all three treatment levels. In contrast, the RSD treatment clearly eliminated the ${\text{NO}}_{3}^{-}$-N in soil. Additionally, RSD treatments reduced the ${\text{NO}}_{3}^{-}$-N content in the soil to a lower level within 7 days. However, the results of the CK treatment showed that flooding alone could not eliminate the ${\text{NO}}_{3}^{-}$-N accumulated in the soil (Figure 1D).

### Effects of Tomato Planting on Soil Properties and Plant Biomass

After the tomato planting, the pH was lower and the ${\text{NH}}_{4}^{+}$-N and DOC contents were significantly higher in the ALH group than in the other groups. Additionally, in the ALH group, the ${\text{NO}}_{3}^{-}$-N content reached up to 279.5 mg kg^–1^, second only to the CK group (Supplementary Figures S1a–d). The results of ${\text{NH}}_{4}^{+}$-N analysis showed that MS, RS, and AL treatments showed similar trends; 5% addition of the substrate yielded significantly higher ${\text{NH}}_{4}^{+}$-N levels than 0.5% and 2% substrate (Supplementary Figure S1b). Analysis of DOC contents yielded similar results (Supplementary Figure S1c). In soil samples with MS and RS treatments, the ${\text{NO}}_{3}^{-}$-N contents tended to decrease as the amount of added substrate increased; the opposite trend was observed in soils treated with AL (Supplementary Figure S1d).

Leaf dry weight decreased in the following order, with significant (*P* < 0.05) differences represented by “>”: MSL > MSM > MSH, RSL > RSM > RSH, ALL > ALM > ALH, and MSL > ALL > RSL. Stem and root dry weight showed the same results in MS and AL treatments. However, in RS treatments, the results of stem and root dry weight were not significantly different at the three input levels. After cultivation for 60 days, the plant height and stem diameter in the MSL group were notably higher than in the other treatments. Additionally, plant height and stem diameter were 36.9% and 23.1% greater, respectively, than in the CK group (Supplementary Table S1).

### Changes in Bacterial Microbial Community Structure During RSD Treatment

#### Fluctuation of Bacterial Population

**FIGURE 2:**
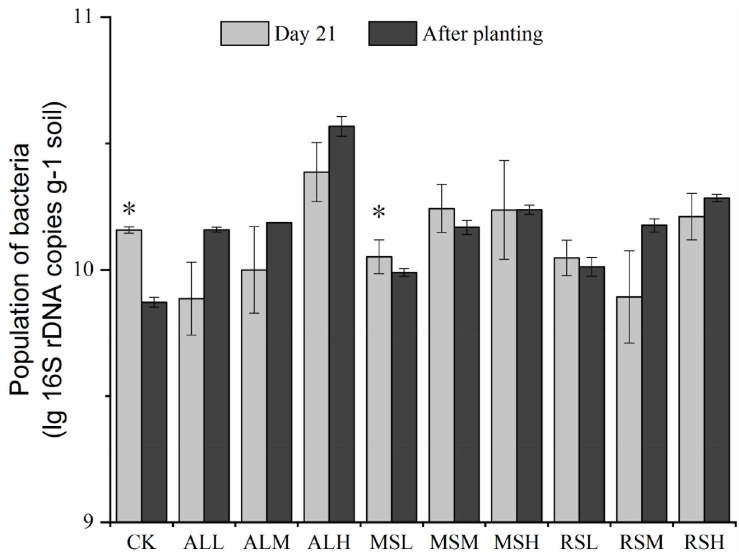
Determination of bacterial populations in samples from the 10 treatments at different time points. Error bars represent standard errors of the means of three replicates. ^∗^*P* < 0.05.

From days 7 to 14, bacterial 16S rRNA gene copies in the CK, MSM, MSH, RSL, RSH, and ALM groups varied significantly (*P* < 0.05, Supplementary Figure S2). However, from day 21 to after tomato planting, only the bacterial 16S rRNA gene copies in the CK and MSL groups were significantly altered (*P* < 0.05). Additionally, after tomato planting, the bacterial 16S rRNA gene copies in all RSD treatments were higher than those in the CK group. For all three substrate treatments, the population of bacteria in the treatment with 5% added substrate was clearly higher than those in treatments with 0.5% and 2% added substrate (Figure 2).

#### Soil Bacterial Composition

**FIGURE 3:**
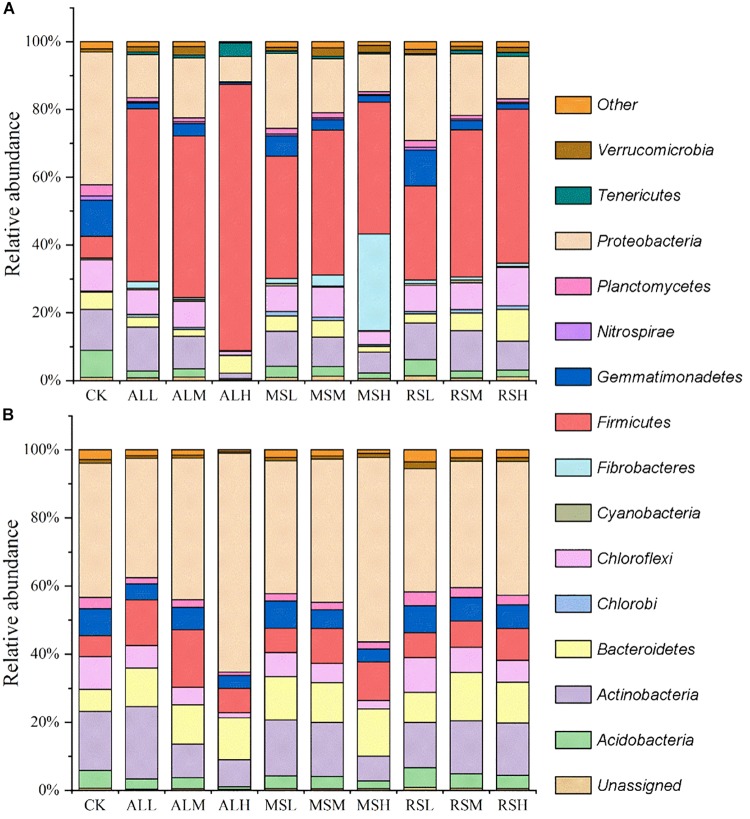
Relative abundances (%) of individual taxonomic groups of the bacterial phyla present in samples from the 10 treatments. **(A,B)** Day 21 and after tomato planting, respectively. Bacterial phyla with relative abundances lower than 1% in all the treatments were clustered as “other.”

During soil treatment, the dominant phyla across all samples were *Acidobacteria*, *Actinobacteria*, *Bacteroidetes*, *Fibrobacteres*, *Firmicutes*, *Nitrospirae*, and *Proteobacteria* (Figure 3A and Supplementary Figures S3a,b). Compared with the CK group, the relative abundance of *Firmicutes* phyla increased in all RSD treatments, and the higher substrate addition improved the relative abundance of *Firmicutes*. On days 7 and 14, there were negative relationships between the relative abundance of *Firmicutes* and the C/N ratio of the substrate. Additionally, on day 21, the relative abundance of *Firmicutes* in all RSD treatments tended to be the same, except for that in the ALH group. The bacterial community composition in all treatments tended to become more and more similar after planting of the tomatoes (Figure 3B). The relative abundance of *Proteobacteria* in all treatments was higher than 35%, making this the dominant phylum after tomato planting (Figure 3B).

The bacterial community structure changed in the soil over time, the initially dominant genera of bacteria gradually disappeared, and the relative abundances of the various genera became balanced (Supplementary Figures S4a–c). At the end of soil incubation (day 21), the ALH treatment significantly increased the relative abundances of *Caloramator*, *Oxobacter*, and *Sedimentibacter* compared with that in other treatments. Moreover, the relative abundances of *Clostridium* and *Coprococcus* were notably higher for all RSD treatments than those in the CK treatment, which were lower than 1% within 21 days (Table 2). During soil incubation, compared with 5% substrate addition, 0.5% substrate addition distinctly increased the relative abundances of *Flavisolibacter* and *Symbiobacterium* in the MS and AL treatments (Supplementary Figures S4a–c). On day 21, the highest relative abundances of *Bacillus* and *Desulfosporosinus* were found in the AL treatments, and lower substrate amounts were associated with higher relative abundances of *Bacillus* and *Desulfosporosinus*. For *Kaistobacter* spp., the relative abundance was significantly higher in the CK group than in all RSD treatments. After tomato planting, the relative abundances of *Stenotrophomonas*, *Pseudomonas*, and *Brevundimonas* were obviously higher in the ALH group than in the other groups. The relative abundance of *Bacillus* decreased in the following order, where “>” represents a significant difference (*P* < 0.05) and “=” represents no significant difference (*P* > 0.05): MSL > MSH, RSL > RSH, ALL > ALH, ALL > MSL = RSL > CK. The relative abundance of *Clostridium* in CK, MSL, RSL, and RSM groups was clearly lower than that in other treatments, with that in the ALM group showing the highest abundance. The relative abundances of *Flavisolibacter* in the MSL and RSM groups reached as high as 5.0% and 4.3%, respectively, showing significantly higher levels than in other treatments. In the *Acidobacteria* phylum, compared with 5% and 2% substrate addition, 0.5% substrate addition distinctly increased the relative abundances of *Streptomyces* in the MS, RS, and AL treatments (Table 3 and Supplementary Figure S4d).

**TABLE 2:** Relative abundances of dominant functional genera and pathogenic genera of bacteria and fungi during RSD treatment.

	**Bacteria**				**Fungi**		
	***Firmicutes* (%)**				***Ascomycota* (%)**		
	***Bacillus***	***Clostridium***	***Coprococcus***	***Desulfosporosinus***	***Aspergillus***	***Fusarium***	***Pseudallescheria***
**Day 7**							
CK	3.14 ± 0.09g^a^	0.90 ± 0.09g	0.54 ± 0.05de	0.09 ± 0.01e	49.90 ± 3.07a	2.85 ± 0.17bc	0.89 ± 0.04a
ALL	10.05 ± 0.75cd	3.76 ± 0.50ef	1.78 ± 0.19d	1.13 ± 0.10cd	50.80 ± 7.24a	0.94 ± 0.20c	3.37 ± 1.84a
ALM	18.60 ± 1.24a	8.30 ± 0.99c	6.17 ± 0.37b	2.05 ± 0.35ab	9.10 ± 1.76d	2.34 ± 0.39c	2.99 ± 0.15a
ALH	7.71 ± 0.44def	21.62 ± 1.90a	3.59 ± 0.55c	1.05 ± 0.21cd	2.47 ± 0.25d	3.11 ± 2.46bc	2.95 ± 0.78a
MSL	6.22 ± 0.86efg	1.42 ± 0.89fg	0.92 ± 0.17de	0.18 ± 0.02e	32.24 ± 1.98bc	2.17 ± 0.27c	1.07 ± 0.07a
MSM	14.35 ± 2.28b	7.27 ± 1.49cd	5.23 ± 0.76b	1.62 ± 0.22bc	31.31 ± 5.20bc	7.26 ± 1.75b	1.06 ± 0.19a
MSH	10.07 ± 1.25cd	15.24 ± 0.38b	7.56 ± 0.55a	2.78 ± 0.66a	4.87 ± 0.80d	15.95 ± 3.29a	2.95 ± 1.59a
RSL	4.58 ± 0.25fg	0.63 ± 0.08g	0.48 ± 0.02e	0.10 ± 0.01e	42.78 ± 5.23ab	1.85 ± 0.08c	1.31 ± 0.11a
RSM	7.89 ± 0.20cde	1.36 ± 0.09fg	1.62 ± 0.22de	0.33 ± 0.04de	49.10 ± 10.60a	1.25 ± 0.30c	2.43 ± 0.90a
RSH	11.11 ± 0.79c	5.22 ± 0.41de	5.89 ± 0.34b	1.37 ± 0.12bc	26.82 ± 1.36c	2.73 ± 0.18bc	1.90 ± 0.10a
**Day 14**							
CK	3.69 ± 0.06bc	0.87 ± 0.03d	0.55 ± 0.04c	0.11 ± 0.01e	52.01 ± 3.12a	2.35 ± 0.49a	1.11 ± 0.21a
ALL	13.98 ± 1.66a	3.81 ± 0.25d	0.89 ± 0.08c	0.77 ± 0.02c	31.71 ± 3.51b	0.08 ± 0.040e	7.03 ± 2.13a
ALM	2.86 ± 0.39c	23.15 ± 0.28a	3.45 ± 0.33b	1.56 ± 0.16ab	0.10 ± 0.03d	0.50 ± 0.27de	0.05 ± 0.02a
ALH	4.18 ± 1.70bc	14.02 ± 6.06bc	7.28 ± 1.70a	0.61 ± 0.08cd	0.54 ± 0.22d	0.06 ± 0.03e	18.94 ± 15.77a
MSL	5.65 ± 0.19bc	1.45 ± 0.16d	0.80 ± 0.07c	0.34 ± 0.10cde	42.53 ± 1.13ab	1.35 ± 0.08bc	2.53 ± 0.59a
MSM	4.50 ± 0.65bc	9.81 ± 0.52bcd	2.40 ± 0.08bc	1.30 ± 0.11b	33.11 ± 5.59b	1.67 ± 0.12b	5.11 ± 2.19a
MSH	4.07 ± 0.92bc	16.59 ± 6.02ab	3.37 ± 0.86b	1.75 ± 0.30a	2.37 ± 0.22d	0.95 ± 0.15cd	3.07 ± 1.24a
RSL	4.22 ± 0.25bc	0.81 ± 0.06d	0.48 ± 0.08c	0.09 ± 0.01e	36.50 ± 5.10b	1.80 ± 0.30ab	3.25 ± 0.74a
RSM	6.66 ± 1.04^b^	1.79 ± 0.11d	1.34 ± 0.10c	0.30 ± 0.03de	15.46 ± 6.07c	0.48 ± 0.10de	26.48 ± 23.88a
RSH	5.22 ± 1.07bc	7.55 ± 0.63cd	2.12 ± 0.20bc	1.21 ± 0.26b	4.73 ± 0.57d	0.18 ± 0.03e	7.15 ± 3.49a
**Day 21**							
CK	1.49 ± 0.03ef	0.74 ± 0.07f	0.15 ± 0.02d	0.05 ± 0.01e	47.63 ± 10.43a	2.75 ± 0.44a	0.75 ± 0.17e
ALL	7.52 ± 0.58a	6.37 ± 0.06b	1.88 ± 0.07b	0.70 ± 0.04a	9.50 ± 1.79cde	0.06 ± 0.02d	6.70 ± 0.27de
ALM	2.15 ± 0.09cde	7.81 ± 0.75a	1.51 ± 0.14bc	0.67 ± 0.08a	10.00 ± 6.06bcde	0.07 ± 0.01d	19.88 ± 6.44c
ALH	1.19 ± 0.18f	4.50 ± 0.88c	5.09 ± 0.51a	0.12 ± 0.03e	0.42 ± 0.19e	0.02 ± 0.01d	87.03 ± 4.92a
MSL	2.89 ± 0.32bc	2.99 ± 0.15e	1.65 ± 0.10bc	0.24 ± 0.02d	24.24 ± 2.34b	0.81 ± 0.11bc	9.43 ± 1.50cde
MSM	1.45 ± 0.18ef	5.06 ± 0.12c	1.39 ± 0.20bc	0.54 ± 0.04b	15.61 ± 3.74bcd	1.36 ± 0.34b	9.14 ± 4.37cde
MSH	2.37 ± 0.19cd	7.03 ± 0.15ab	4.64 ± 0.56a	0.43 ± 0.01c	1.57 ± 0.41de	0.51 ± 0.21cd	63.76 ± 4.82b
RSL	1.98 ± 0.15def	4.22 ± 0.36cd	1.05 ± 0.06bc	0.39 ± 0.04c	22.91 ± 1.98bc	1.12 ± 0.14b	5.59 ± 0.55de
RSM	3.39 ± 0.30b	3.22 ± 0.02de	1.46 ± 0.07bc	0.35 ± 0.01c	12.75 ± 4.09bcde	0.12 ± 0.01d	15.87 ± 3.07cd
RSH	1.68 ± 0.09def	4.47 ± 0.05c	1.00 ± 0.08c	0.55 ± 0.01b	6.78 ± 2.41de	0.13 ± 0.03d	6.04 ± 1.55de

*^*a*^Data are mean ± standard error, and different lowercase letters indicate significant differences between treatments (*P* < 0.05).*

**TABLE 3:** Relative abundances of dominant functional genera and pathogenic genera of bacteria and fungi in tomato rhizosphere soils.

	**Bacteria**				**Fungi**		
	***Firmicutes* (%)**		***Acidobacteria* (%)**	***Bacteroidetes* (%)**	***Ascomycota* (%)**		
		
	***Bacillus***	***Clostridium***	***Streptomyces***	***Flavisolibacter***	***Aspergillus***	***Fusarium***	***Arthrographis***
CK	1.82 ± 0.21c^a^	0.47 ± 0.03e	1.64 ± 0.02c	0.30 ± 0.03e	52.91 ± 3.84a	2.35 ± 0.74a	0.70 ± 0.08c
ALL	5.06 ± 0.12a	0.99 ± 0.02d	3.55 ± 0.41a	3.05 ± 0.44b	0.30 ± 0.02d	0.08 ± 0.01b	0.43 ± 0.11c
ALM	2.17 ± 0.13c	4.40 ± 0.30a	0.82 ± 0.02e	0.71 ± 0.13de	2.64 ± 0.68bcd	0.50 ± 0.02b	25.53 ± 2.09b
ALH	1.94 ± 0.24c	1.03 ± 0.10d	0.48 ± 0.10e	0.26 ± 0.04e	0.80 ± 0.11cd	0.36 ± 0.16b	62.74 ± 3.77a
MSL	3.05 ± 0.22b	0.30 ± 0.03e	2.59 ± 0.11b	5.00 ± 0.36a	5.51 ± 0.96b	0.78 ± 0.18b	1.73 ± 0.17c
MSM	1.62 ± 0.01c	2.40 ± 0.13c	0.97 ± 0.04de	1.91 ± 0.01c	5.13 ± 0.64bc	1.21 ± 0.11b	0.32 ± 0.05c
MSH	2.19 ± 0.30c	3.62 ± 0.19b	0.53 ± 0.05e	1.32 ± 0.09cd	1.87 ± 0.12bcd	0.82 ± 0.10b	0.15 ± 0.04c
RSL	2.89 ± 0.22b	0.32 ± 0.01e	1.48 ± 0.34cd	1.37 ± 0.03cd	1.77 ± 0.26bcd	0.98 ± 0.71b	0.55 ± 0.16c
RSM	2.75 ± 0.09b	0.27 ± 0.02e	0.58 ± 0.10e	4.27 ± 0.66a	0.93 ± 0.08cd	0.25 ± 0.08b	2.52 ± 0.27c
RSH	2.00 ± 0.08c	1.22 ± 0.11d	0.56 ± 0.02e	1.65 ± 0.14c	3.43 ± 0.60bcd	1.05 ± 0.05b	0.47 ± 0.07c

*^*a*^Data are mean ± standard error, and different lowercase letters indicate significant differences between treatments (*P* < 0.05).*

#### PCoA of Bacterial Communities

**FIGURE 4:**
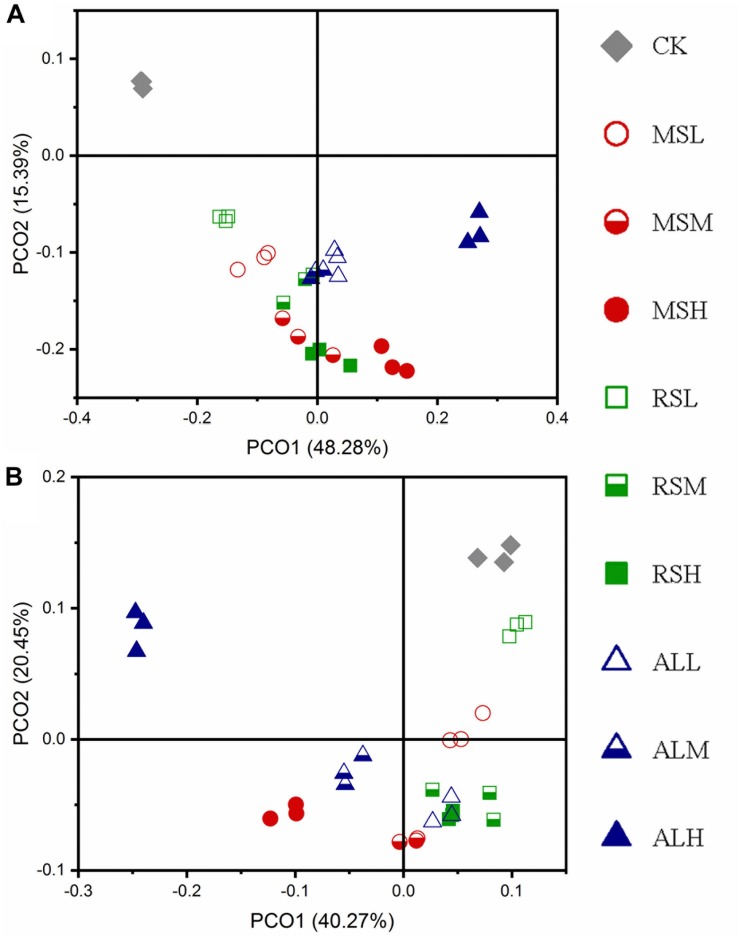
Principal coordinate analysis (PCoA) for the dissimilarity of the bacteria microbial communities in the different soils. **(A,B)** Day 21 and after tomato planting, respectively. The PCoA of bacterial was based on weighted-unifrac indexes.

During the 3-week study period, PCoA showed that RSD treatments significantly (*P* = 0.001) influenced soil bacterial community structures compared with the CK treatment. During soil treatment, the bacterial community structures tended to diverge first and then converge (Figure 4A and Supplementary Figures S5a,b). Compared with the bacterial community structure after RSD treatments, the microbial community structure in the soil was changed after tomato planting (Figures 4A,B).

### Changes in Fungal Microbial Community Structure During RSD Treatment

#### Fluctuation of Fungal Population

**FIGURE 5:**
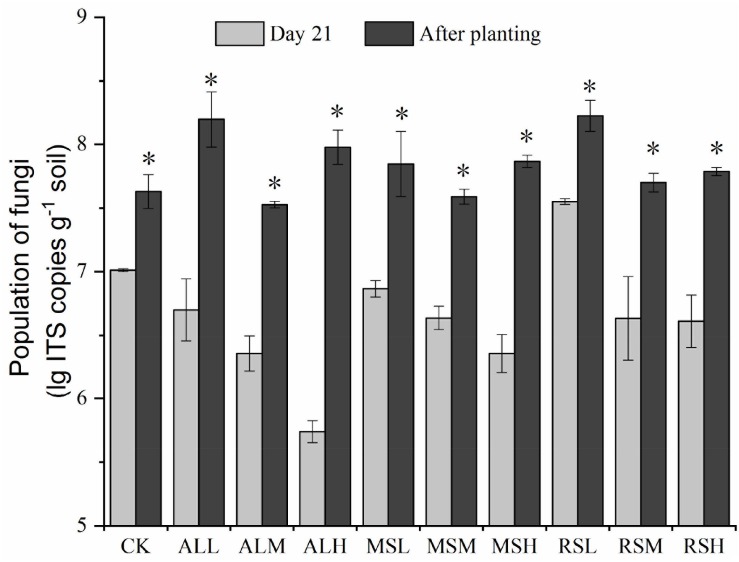
Determination of fungal populations from the 10 treatments at different time points. Error bars represent the standard errors of the means of three replicates. ^∗^*P* < 0.05.

On day 7, the copies of fungal ITS genes decreased in the following order: 0.5% > 2% > 5% (*P* < 0.05). On day 14, the numbers of fungal ITS genes did not differ significantly in all groups except for the CK, MSM, MSH, and RSL groups as compared with day 7 (Supplementary Figure S6). However, compared with that before tomato planting, tomato planting notably increased the copies of fungal ITS genes in the soil (*P* < 0.05; Figure 5).

#### Soil Fungal Composition

**FIGURE 6:**
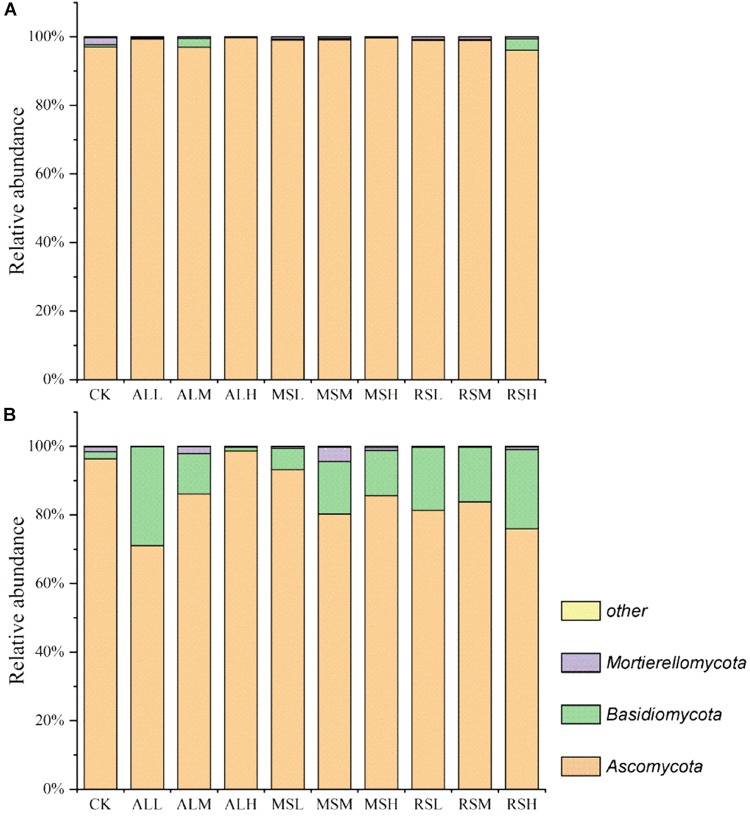
Relative abundances (%) of individual taxonomic groups of the fungal phyla present in samples from the 10 treatments. **(A,B)** Day 21 and after tomato planting, respectively. The fungal phyla with a relative abundance lower than 1% in all the treatments were clustered as “other.”

During soil treatment, *Ascomycota* was the dominant phylum in all soils, with a relative abundance higher than 90% (Figure 6A and Supplementary Figures S7a,b). However, after planting tomatoes, although the relative abundance of *Ascomycota* was the highest, the relative abundance of *Basidiomycota* was increased in all soils (Figure 6B).

Specifically, at the fungal genus level, significant variations were observed in relative abundances of dominant genera (Table 2 and Supplementary Figures S8a–c). During soil treatments, the relative abundances of *Aspergillus* and *Fusarium* in the RSD treatments tended to decrease over time. However, by day 21, the relative abundance of *Aspergillus* in CK still remained high (47.6%), and the relative abundances of *Aspergillus* and *Fusarium* were significantly higher than those in all RSD treatments. Notably, at the end of soil treatment, compared with CK treatment, the ALH and MSH treatments clearly reduced the relative abundances of *Acremonium*, also leading to a dramatic increase in the relative abundance of *Pseudallescheia*. After tomato planting, the relative abundance of *Aspergillus* was significantly higher in the CK group than in all RSD treatments, and its relative abundance in the CK group exceeded 50% of all fungi. Similar to *Aspergillus*, the relative abundance of *Fusarium* was the highest in the CK group, and there were no clear differences between all RSD treatments (Table 3). For the *Arthrographis* genera, the relative abundances in the ALM and ALH groups were obviously higher than in the other groups, reaching up to 25.5% and 62.7%, respectively (Table 3 and Supplementary Figure S8d).

#### PCoA of Fungal Communities

**FIGURE 7:**
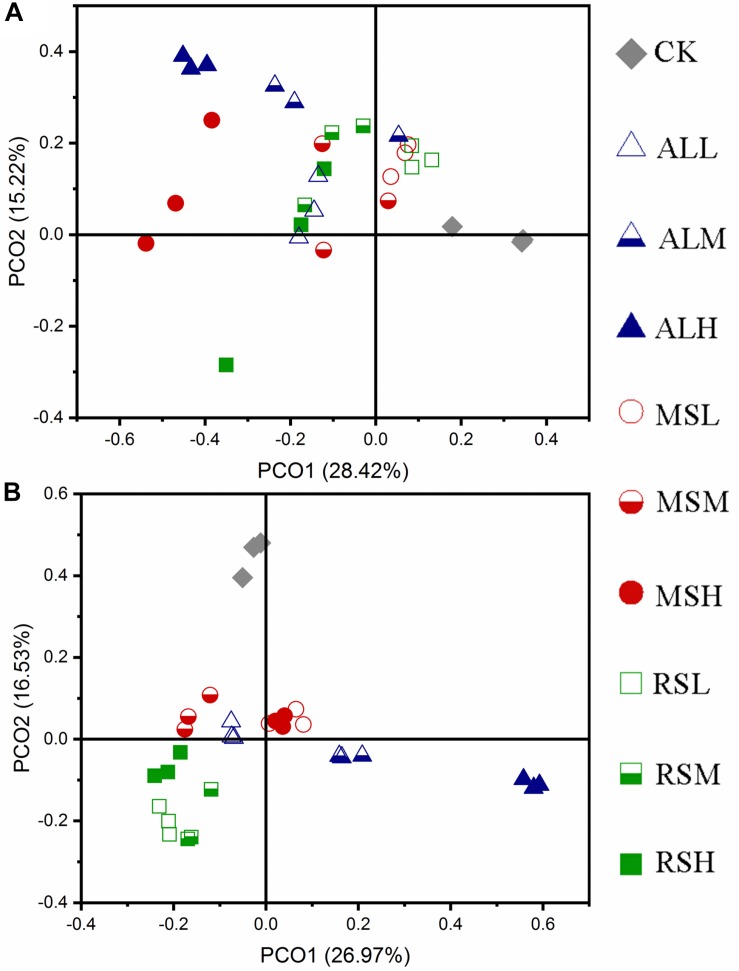
Principal coordinate analysis for the dissimilarities of fungal microbial communities in different soils. **(A,B)** Day 21 and after tomato planting, respectively. PCoA of fungi based on the bray_curtis index.

During soil treatments, compared with the CK treatment, the fungal community structures of RSD treatments gradually changed over time. When the C/N ratio and the added amount of the substrate differed, the response of the fungal community also differed (Figure 7A and Supplementary Figures S9a,b). After tomato planting, PCoA showed that the CK and ALH treatments were clearly (*P* = 0.001) separated from the other groups. However, the other eight groups clustered together (*P* = 0.1); that is, the fungal community structures in the MSL, MSM, MSH, RSL, RSM, RSH, ALL, and ALM groups tended to be similar after tomato planting (Figure 7B).

## Discussion

The C/N ratio is a key factor in RSD treatment (Liu et al., 2016; Shrestha et al., 2018). The results of ${\text{NH}}_{4}^{+}$-N content analysis showed that higher C/N ratios were associated with lower ${\text{NH}}_{4}^{+}$-N contents when the substrate input amount and treatment time were the same. Thus, the high C/N ratio may limit ${\text{NH}}_{4}^{+}$-N accumulation (Khalil et al., 2005). Because the amount of nitrogen consumed for carbon assimilation is constant, there will be less N remaining after carbon assimilation in the presence of a high C/N ratio (Rosskopf et al., 2015). In contrast, Butler (2014) observed that the high C/N ratio of organic matter may cause loss of nitrogen. Moreover, previous studies (Mary et al., 1996) have also shown that organic amendment with a high C/N ratio is more conducive to immobilization of nitrogen and that mineralization of low C/N ratio organic residues is higher than that of high C/N ratios during decomposition. In addition to mineralization, anaerobic conditions and high soil temperatures may also affect the activities of nitrifying soil bacteria (Butler et al., 2014; Rosskopf et al., 2015), leading to accumulation of ${\text{NH}}_{4}^{+}$-N. At the end of the RSD treatment, the ${\text{NH}}_{4}^{+}$-N content in the ALH group (with the lowest C/N ratio) was as high as 979 mg kg^–1^, which may result in ammonium toxicity. The results of ${\text{NO}}_{3}^{-}$-N analysis showed that RSD treatments could effectively eliminate ${\text{NO}}_{3}^{-}$-N accumulated in the soil; lower C/N ratios were associated with better effects. Increased DOC contents in the soil are conducive to the growth of soil microorganisms (Martínez-García et al., 2018), including bacteria related to denitrification. In our study, we found that when the substrate input amount and the treatment time were the same, the DOC content in the soil decreased as the C/N ratio increased. Therefore, low C/N ratios could provide more DOC for denitrifying bacteria and enhance denitrification, resulting in decreased ${\text{NO}}_{3}^{-}$-N contents in the soil. An anaerobic environment also promotes this process. The products of denitrification are mainly N_2_O and N_2_, and release of N_2_O causes a greenhouse effect (Ciarlo et al., 2006). Thus, in order to reduce nitrogen losses, the release of greenhouse gases (N_2_O), and the toxic effects of high ammonia nitrogen levels on plants, a C/N ratio about 30–35 should be selected for RSD amendments.

In this study, soil microbial communities were distinctly changed during RSD treatments, and this change is one of the mechanisms of RSD (Huang et al., 2016; Zhao et al., 2018). During the RSD treatments, the microbial diversity (α and β) in each treated soil decreased with both bacteria and fungi (Huang et al., 2019). This is due to the fact that most aerobic bacteria and fungi are inhibited from growing in an anaerobic environment. The relative abundance of anaerobic bacteria, such as the *Firmicutes*, rises sharply and becomes the dominant bacteria in each treatment.

In bacteria, during the RSD treatment, the products *N*-acetyl-β-glucosaminidase and β-glucosidase from *Flavisolibacter* (Yoon and Im, 2007) (phylum *Bacteroidetes*) and the β-*N*-acetylhexosaminidase and exo-type β-glycosidase produced by *Symbiobacterium* (phylum *Firmicutes*) (Suzuki et al., 1988; Ohno et al., 2000; Ogawa et al., 2006) possibly contributed to the degradation of organic material. In our study, the relative abundances of *Flavisolibacter* and *Symbiobacterium* in the MSL, RSM, and ALL groups were higher than those in the CK group, and a low level of substrate addition (0.5%) resulted in significantly higher abundances than a high level of substrate addition (5%) in the MS and AL treatment groups. Compared with days 7 and 14, on day 21, *Flavisolibacter* was significantly reduced in the MSL, RSL, RSM, and ALL groups. There were two possible reasons for this result. First, *Flavisolibacter* is an aerobic bacterium (Kim et al., 2018); greater substrate addition would result in more favorable formation of an anaerobic environment (Wen et al., 2015), and therefore, a high addition amount was not conducive to the growth of *Flavisolibacter*. Second, during the early stage of cultivation, residual oxygen in the soil and dissolved oxygen in the water could provide conditions for the survival of *Flavisolibacter*; *Flavisolibacter* increased rapidly in the presence of a sufficient carbon source. Therefore, lack of oxygen during the later period led to a decrease in the abundance of *Flavisolibacter*.

Previous reports have demonstrated that organic acids produced in RSD treatment have important inhibitory effects on pathogens (Momma et al., 2006; Huang et al., 2014). Most of the producers of these organic acids belong to *Firmicutes*, such as *Sedimentibacter*, *Desulfosporosinus*, *Oxobacter*, *Clostridium*, and *Coprococcus*. *Sedimentibacter* (Imachi et al., 2016), *Desulfosporosinus* (Stanley and Southam, 2018), *Oxobacter* (Bengelsdorf et al., 2015), *Clostridium* (Braun et al., 1981), and *Coprococcus* (Holdeman and Moore, 1974) are effective producers of acetic acids, butyric acid, and propionic acid. During RSD treatment, some genera of *Firmicutes* showed significant changes compared with the CK group, and the relative abundances of organic acid producers in the CK group were less than 1% throughout the soil treatment period. However, after RSD treatments, the relative abundances of organic acid producers increased, particularly in the AL groups, potentially because many *Firmicutes* are anaerobic bacteria and the low C/N ratio is more conducive to the formation of an anaerobic environment (Liu et al., 2016). In addition, the higher addition of substrate was related to higher relative abundance of organic acid producers, similar to the results of a study by Wen (2015). Once pathogens are inhibited, toxic organic acids are degraded during the drying process of the soil due to destruction of the anaerobic environment, thereby preventing toxicity during crop cultivation. In addition to producing these organic acids to inhibit pathogens, *Desulfosporosinus* and *Bacillus* have been shown to produce sulfide and low-valence metal ions (Fe^2+^, Mn^2+^) (Boone et al., 1995; Stanley and Southam, 2018), respectively, and are also involved in the disinfestation process of RSD (Runia et al., 2014). Notably, during RSD treatment, the relative abundance of *Desulfosporosinus* reached a maximum of only 0.1% in the CK group, which could also explain why there was a lack of pathogen inhibition. After tomato planting, the relative abundances of genera with disease resistance, phosphorus solubilization, or nitrogen fixation ability, such as *Bacillus* (Ji et al., 2008; Jeong et al., 2012), *Clostridium*, *Streptomyces* (Watve et al., 2001), and *Flavisolibacter* (Mpanga et al., 2018), were significantly higher in the RSD treatment with 0.5% substrate addition than in the CK group, corresponding to the results of tomato plant biomass.

Reductive soil disinfestation treatments clearly reduced the levels of fungal pathogens. *Penicillium* and *Fusarium* can cause plant disease (Larkin and Fravel, 1998; Meng et al., 2017). Additionally, the abundances of *Penicillium* and *Fusarium* in some RSD treatments were higher than those in the CK group on day 7; notably, addition of more substrate increased the abundances of *Penicillium* and *Fusarium*. Moreover, over time, the abundances of *Penicillium* and *Fusarium* in the RSD treatments gradually decreased. However, the abundance of *Penicillium* in the CK group also decreased with time, indicating that the CK treatment could effectively inhibit *Penicillium* under pure flooding and anaerobic conditions. For *Fusarium*, pure flooding did not inhibit *Fusarium*; however, the RSD treatments significantly reduced the abundance of *Fusarium*. On day 21, the relative abundances of *Fusarium* in RSD treatments, except the MSM and RSL treatments, were reduced to less than 1%, and those in AL treatment groups were reduced to below 0.1%. This confirmed the findings of Liu (2016), demonstrating that lower C/N ratios were associated with better disinfestation efficiencies. Many previous studies have also shown that RSD can effectively suppress *Fusarium* (Larkin and Fravel, 1998; Huang et al., 2014; Meng et al., 2017). Notably, for the pathogen *Aspergillus* (Wang et al., 2017), by day 21, RSD treatments effectively suppressed *Aspergillus*, and the relative abundance in the CK group was clearly higher than those in all RSD treatment groups, consistent with the results of Huang et al. (2016). Furthermore, after tomato planting, the abundances of *Fusarium* and *Aspergillus* in the CK group were dramatically higher than those in the RSD treatment groups, demonstrating that RSD had persistent effects.

Interestingly, some fungi (*Pseudallescheria* before planting, *Arthrographis* after planting) were extremely abundant in the ALH group, both before and after planting. These results could be explained by the high nitrogen concentrations encountered after ALH treatment, which would be suitable for the survival and rapid propagation of these fungi. However, this will reduce the microbial diversity in the soil and is not conducive to soil health.

Reductive soil disinfestation treatments can effectively inhibit soil-borne diseases (Meng et al., 2019), but plant growth is not only dependent on healthy soil, but also inseparable from adequate nutrition. In our study, the dry weights of the leaf, stem, and root of the MSL treatment were significantly higher than in the CK treatment, while the leaf, stem and root dry weights of the ALH and RSH treatments were notably lower than CK treatment. In fact, in terms of pathogenic bacteria, these three treatments have significant inhibitory effects on pathogenic bacteria, but in terms of nitrogen, when the substrate is added at 5%, the low C/N in ALH treatment caused ammonia toxicity. High C/N in the RSH treatment caused a serious deficiency of nitrogen. This has a certain negative impact on the growth of tomatoes. Based on the results of *Penicillium*, *Fusarium*, *Pseudallescheria*, and *Arthrographis*, in order to balance the relationships among pathogens, substrate addition, and disinfestation effects, we must consider the optimal amount of organic materials in the RSD treatments. For example, in soils in which soil-borne diseases are not serious, we can give priority to adding 0.5% substrate and achieving a C/N ratio of about 30:1 (e.g., MS) for the substrate. Additionally, if RS is applied, sufficient nitrogen fertilizer should be added during crop cultivation. In contrast, for soils with more serious soil-borne diseases, lower C/N ratios for the substrate can be selected, and the amount of input can be increased.

## Conclusion

We examined the effects of different RSD treatments on microbial community structures. Notably, the biomasses of tomatoes in the MSL, MSM, and ALL groups were higher than those in the CK group. Corresponding to changes in microbial communities, after tomato planting, the relative abundances of some beneficial bacteria, such as *Bacillus*, *Clostridium*, *Flavisolibacter*, and *Streptomyces*, were significantly increased in the MSL, MSM, and ALL groups compared with those in the CK group. At the same time, the relative abundances of pathogenic bacteria, such as *Fusarium* and *Aspergillus*, were significantly lower than in the CK group. Although pathogen inhibition was better for treatments with higher substrate concentrations, when the C/N ratio was too low, ammonia toxicity occurred in tomato plants, and rapid proliferation of some fungi that were detrimental to plant growth and soil health was observed, particularly for the ALH group. Therefore, in this study, based on our comprehensive analysis of disinfestation effects and tomato growth status, 0.5% substrate addition was found to be suitable for RSD treatment, compared to 2% and 5% substrate addition. However, when the C/N ratio is higher than 33, the addition amount could be appropriately increased. If considering some fungi (e.g., *Pseudallescheria* and *Arthrographis*) that can grow rapidly in a low C/N environment, the optimal substrate C/N ratio may be approximately 30.

## Data Availability Statement

The datasets generated for this study can be found in the NCBI (SRA accession: PRJNA523309).

## Author Contributions

All authors listed have made a substantial, direct and intellectual contribution to the work, and approved it for publication.

## Conflict of Interest

The authors declare that the research was conducted in the absence of any commercial or financial relationships that could be construed as a potential conflict of interest.
