# Supplementary material for: Effect of Different Substrates on Soil Microbial Community Structure and the Mechanisms of Reductive Soil Disinfestation
Source: Front Microbiol. 2019 Dec 11;10:2851. doi: 10.3389/fmicb.2019.02851 (PMC6917583; doi:10.3389/fmicb.2019.02851)
Supplement: Supplementary file 1 [file Table_1.DOCX]

**Title Page**

**Supplementary Material**

**Journal Name**

Frontiers in Microbiology

**Title**

Effect of different substrates on soil microbial community structure and the mechanisms of reductive soil disinfestation

**Author Names**

Xingyan Tan^1,2^·Hongkai Liao^1,3^·Liangzuo Shu^2**^·Huaiying Yao^1,3,4*^

**Author Affiliation**

^1^Ningbo Key Lab of Urban Environment Process and Pollution Control, Ningbo Urban Environment Observation and Research Station—NUEORS, Institute of Urban Environment,Chinese Academy of Sciences, No. 88 Zhong Ke Road, Ningbo 315830, China

^2^School of Life Sciences, Huaibei Normal University /Key Laboratory of Plant resources and Biology of Anhui Province, Huaibei 235000, Anhui, China

^3^Key Lab of Urban Environment and Health, Institute of Urban Environment, Chinese Academy of Sciences, No. 1799 Jimei Road, Xiamen 361021, China

^4^Research Center for Environmental Ecology and Engineering, Wuhan Institute of Technology, Wuhan 430073, China

**Corresponding author**

*Huaiying Yao, E-mail: [hyyao@iue.ac.cn](mailto:hyyao@iue.ac.cn); Tel: +86-0592-6190791; Fax: +86-0592-6190791

**Liangzuo Shu, E-mail: [Shulz69@163.com](mailto:Shulz69@163.com)

Table S1. Determination of the biomass and growth index of tomato plants

| Treatment | Dry weight (g plant^-1^) | | | Plant height  (cm) | Stem diameter  (cm) |
| --- | --- | --- | --- | --- | --- |
|  | Leaf | Stem | Root |  |  |
| CK | 3.745 ± 0.172c^a^ | 2.289 ± 0.082c | 1.400 ± 0.051c | 23.60 ± 0.12d | 0.701 ± 0.018c |
| ALL | 4.720 ± 0.130b | 3.971 ± 0.317b | 1.668 ± 0.058b | 31.07 ± 2.08c | 0.817 ± 0.011b |
| ALM | 1.382 ± 0.197f | 2.159 ± 0.442c | 0.906 ± 0.090e | 28.50 ± 0.92c | 0.669 ± 0.037c |
| ALH | 0.228 ± 0.027g | 0.230 ± 0.032e | 0.227 ± 0.018f | 8.55 ± 0.32e | 0.467 ± 0.038d |
| MSL | 6.349 ± 0.124a | 4.859 ± 0.165a | 2.250 ± 0.039a | 37.40 ± 0.12a | 0.912 ± 0.010a |
| MSM | 4.429 ± 0.162b | 3.315 ± 0.305b | 1.821 ± 0.091b | 34.17 ± 0.87b | 0.796 ± 0.018b |
| MSH | 2.277 ± 0.234e | 1.808 ± 0.268cd | 1.410 ± 0.111c | 29.10 ± 0.64c | 0.732 ± 0.055bc |
| RSL | 3.109 ± 0.034d | 1.854 ± 0.068cd | 1.349 ± 0.037cd | 28.80 ± 0.92c | 0.732 ± 0.018bc |
| RSM | 2.412 ± 0.145e | 1.787 ± 0.074cd | 1.278 ± 0.027cd | 28.45 ± 0.55c | 0.669 ± 0.017c |
| RSH | 1.690 ± 0.070f | 1.238 ± 0.066d | 1.133 ± 0.106d | 24.03 ± 0.90d | 0.648 ± 0.011c |

^a^Data are mean ± standard error, and different lowercase letters indicate significant differences between treatments（*P* < 0.05).





Fig. S1 The soil pH (a), DOC content (c), ammonia nitrogen content (b) and nitrate nitrogen content (d) in after- planting soils. Bars with different letters represent significant differences among the ten treatments at the same time, as determined using Duncan’s tests (*P*< 0.05). Error bars indicate standard errors.





Fig. S2 Determination of bacterial populations in samples from the 10 treatments at different time points. Error bars represent the standard error of the means of three replicates.





Fig. S3 Relative abundances (%) of individual taxonomic group of the bacterial phyla present in samples from the 10 treatments. (a, b) Day 7 and Day 14, respectively. Bacterial phyla with relative abundances lower than 1% in all the treatments were clustered as “other”.


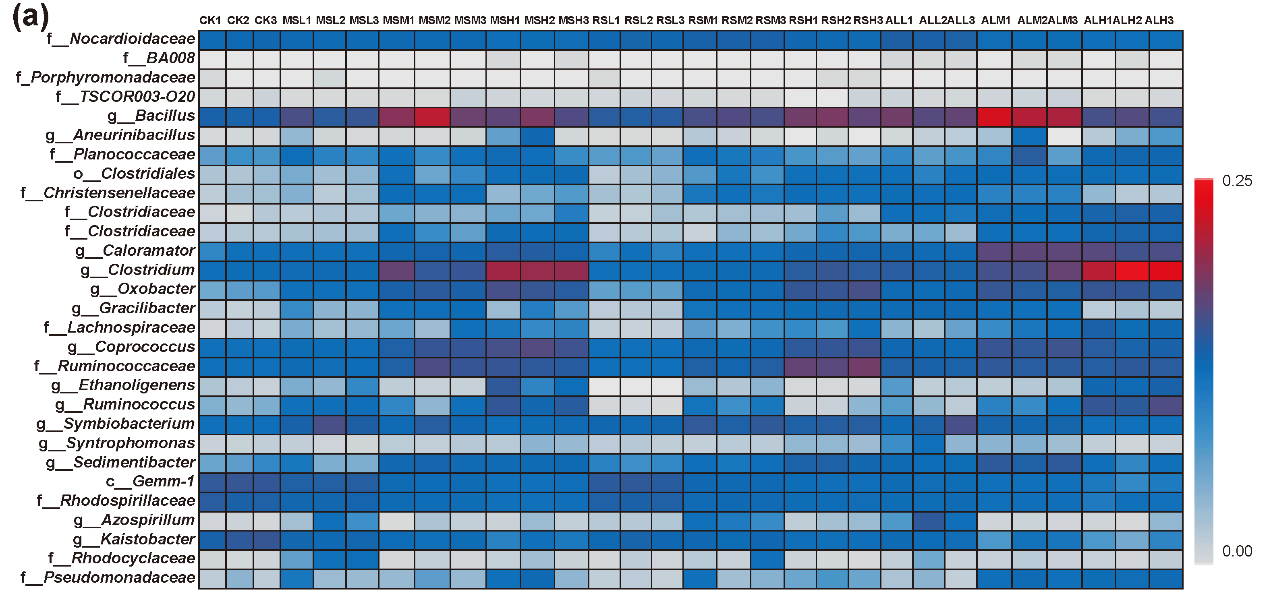


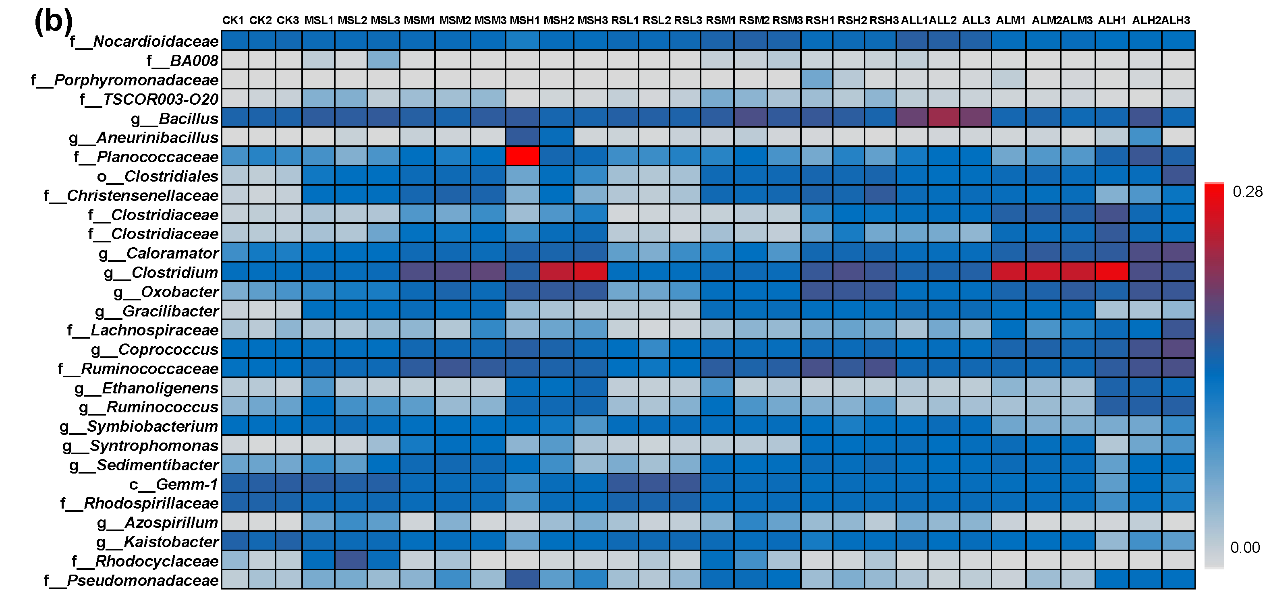


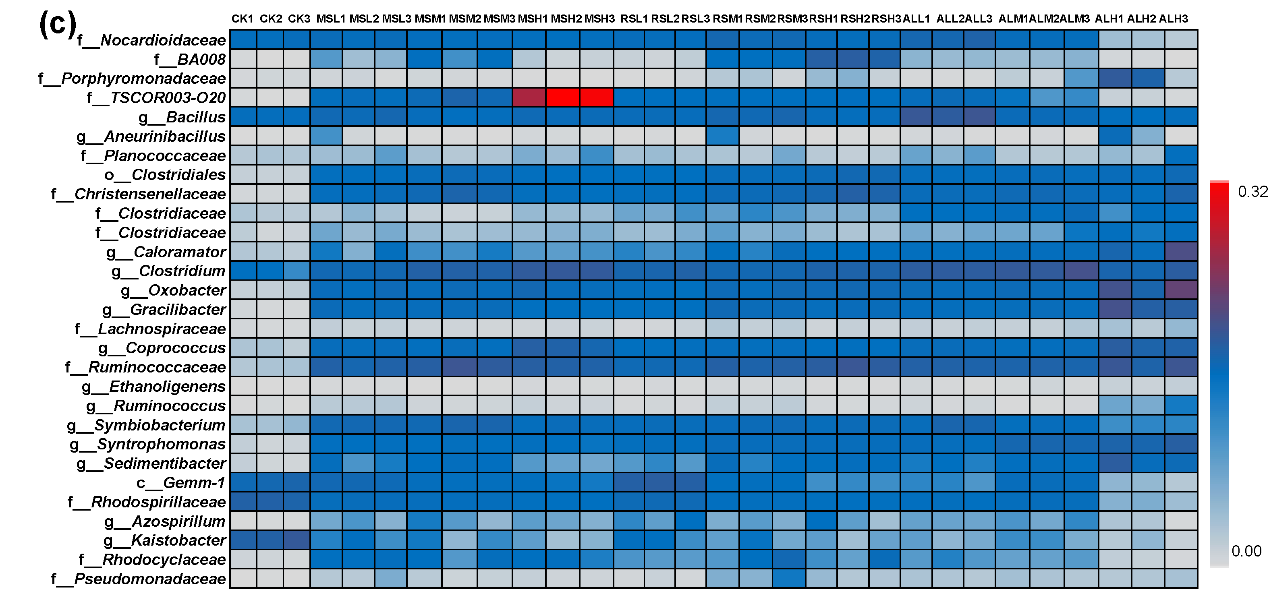


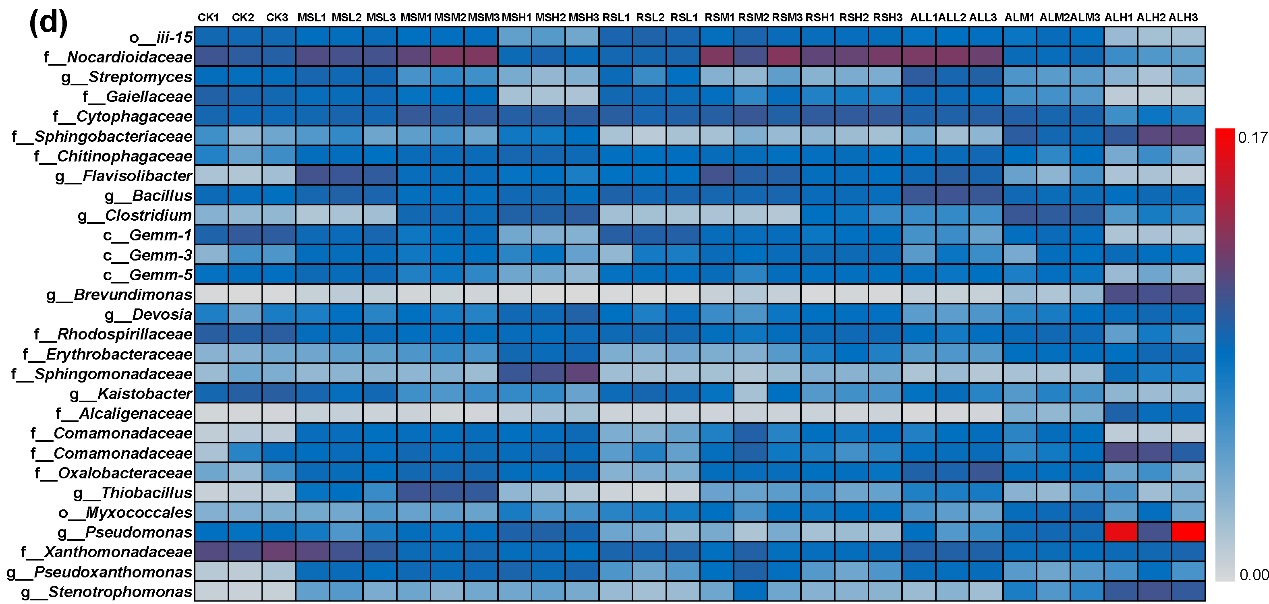


Fig. S4 Heat map illustrating of bacterial composition over time in different soils. (a),(b), (c), and (d) represent day 7, day 14, day 21, and after tomato planting, respectively. The bacterial genera in any of the treatments with relative abundance larger than 5% were listed in the figure during RSD treatment.The bacterial genera in any of the treatments with relative abundance larger than 2% were listed in the figureafter tomato planting.





Fig. S5 Principal coordinate analysis (PCoA) for the dissimilarity of the bacteria microbial communities in the different soils. (a, b) Day 7 and Day 14, respectively. The PCoA of bacterial was based on weighted-unifrac indexes.





Fig. S6 Determination of fungal populations from the 10 treatments at different time points. Error bars represent the standard errors of the means of three replicates.





Fig. S7 Relative abundances (%) of individual taxonomic group of the fungal phyla present in samples from the 10 treatments. (a, b) Day 7 and Day 14, respectively. The fungal phyla with a relative abundance lower than 1% in all the treatments were clustered as “other”


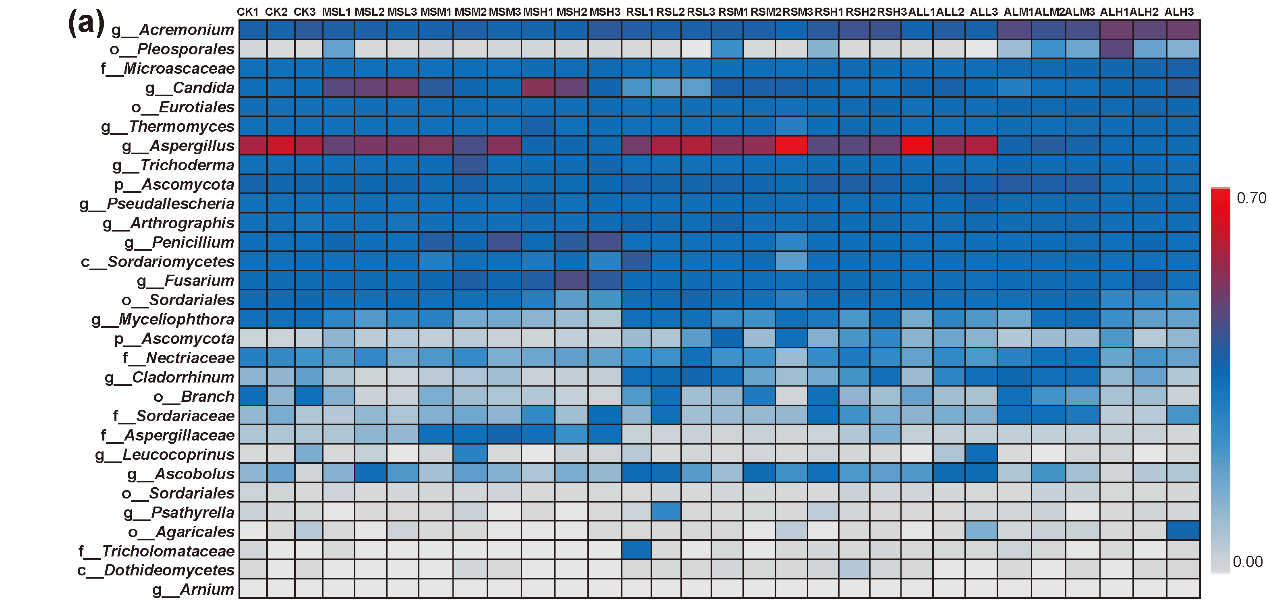


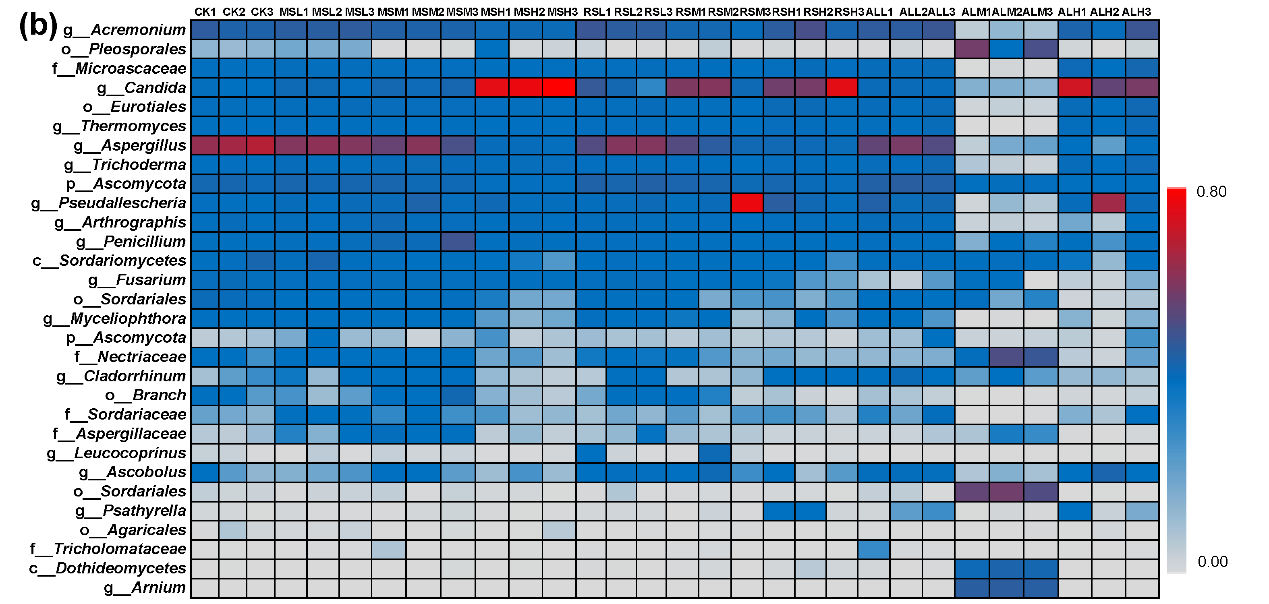


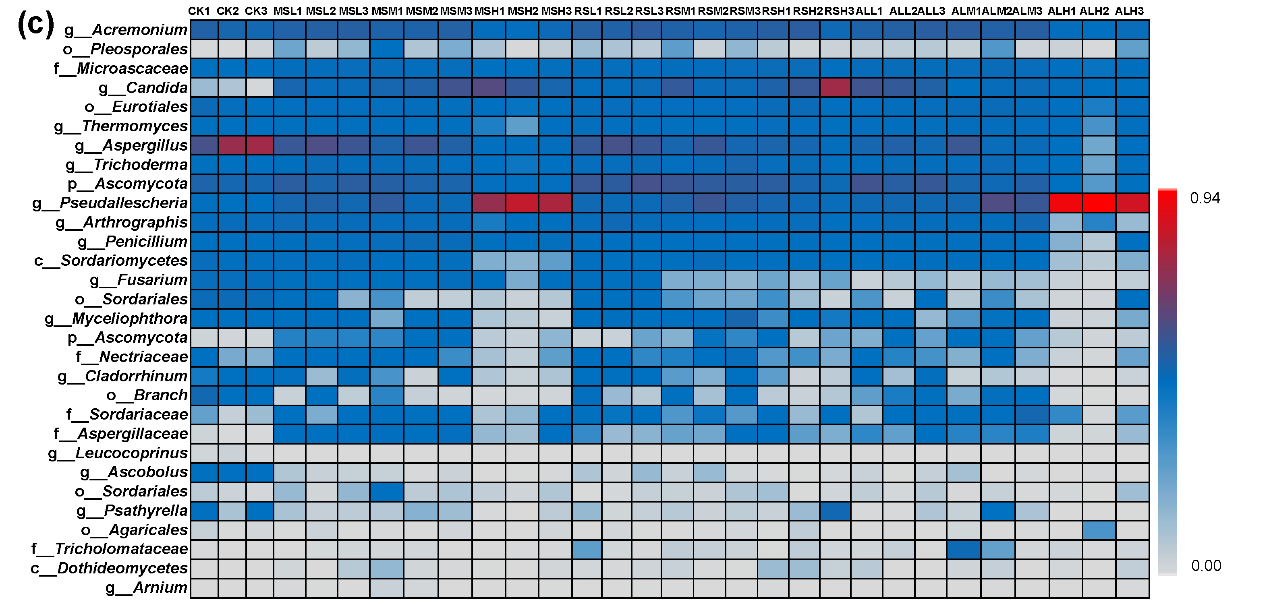


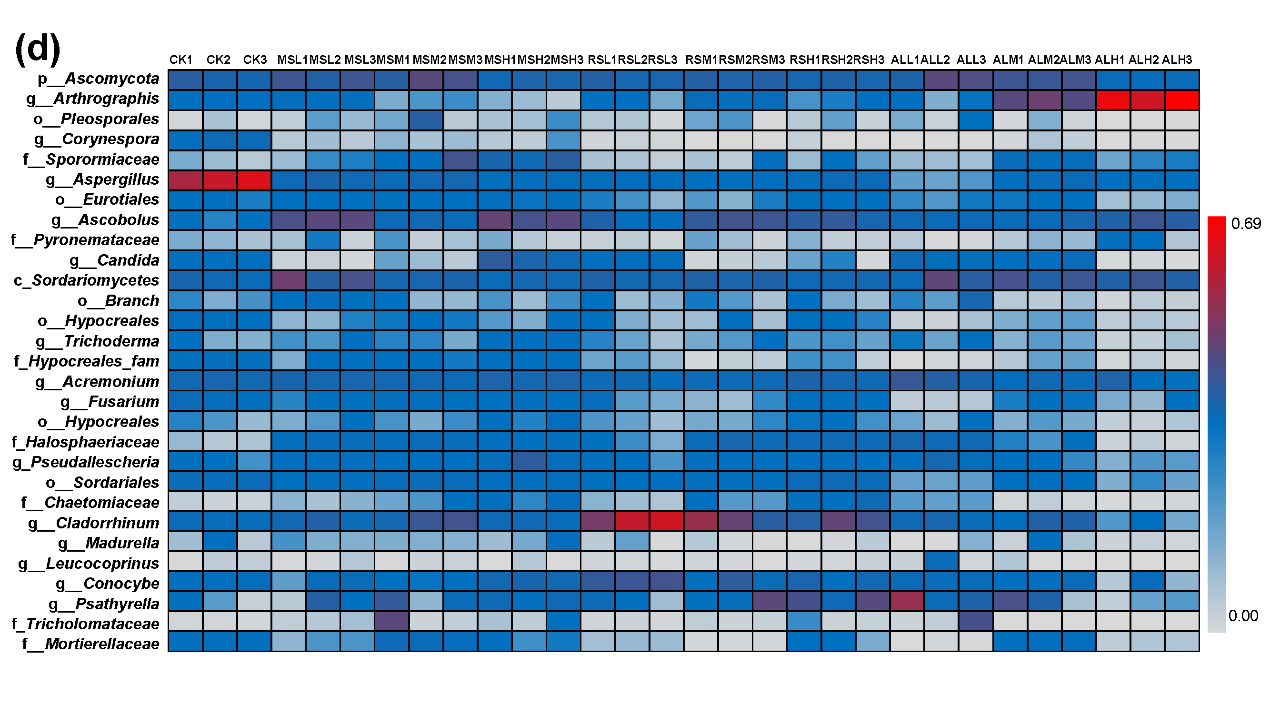
Fig. S8 Heat map illustrating of fungal composition over time in different soils. (a), (b), (c), and (d) represent day 7, day 14, day 21, and after tomato planting, respectively. The fungal genera in any of the treatments with relative abundance larger than 5% were listed in the figure during RSD treatment.The fungal genera in any of the treatments with relative abundance larger than 1% were listed in the figureafter tomato planting.





Fig. S9Principal coordinate analysis (PCoA) for the dissimilarities of the fungal microbial communities in the different soils. (a, b) Day 7 and Day 14, respectively.PCoAof fungi based on thebray_curtis index.
